# Associations Among Interparental Violence, Maternal Depression and Behavioural Difficulties in Preschool‐Aged Children in Jamaica

**DOI:** 10.1002/ijop.70258

**Published:** 2026-08-11

**Authors:** Suzette Kelly‐Williams, Jaipaul L. Roopnarine, Ambika Krishnakumar, Dickson M. M. Ong'ayi

**Affiliations:** ^1^ Missouri State University Springfield Missouri USA; ^2^ Department of Psychology Anton de Kom University of Suriname Paramaribo Suriname; ^3^ Early Childhood Development and Care Center University of Guyana Turkeyen Guyana; ^4^ Department of Human Development and Family Science Syracuse University Syracuse New York USA

**Keywords:** child anxiety/depression, depression, interparental violence

## Abstract

To spotlight women's and children's mental health needs and human rights issues around interparental violence in Caribbean families, this study examined the mediating function of maternal depressive symptoms on associations between interparental violence and children's anxiety/depression, antisocial behaviours, and peer problems. Participants included 152 mothers and their preschool‐aged children residing in the Kingston area of Jamaica. Interparental violence had direct associations with children's antisocial behaviours and peer problems. Maternal depressive symptoms partially mediated the association between interparental violence and child anxiety/depression. Findings point to the importance of considering both interparental violence and maternal depression together in addressing children's mental health difficulties in the broader Caribbean region.

It has been aptly demonstrated that interparental violence and parental depression affect children's socioemotional functioning adversely across cultural communities (Roopnarine et al. 2023; Fong et al. 2019; Yetter 2022). However, much of the research on these two major family risk factors and childhood development has been conducted in high‐income countries with far less attention paid to families in other parts of the world. Data on how interparental violence and depression jointly influence children's socioemotional functioning during the susceptible early childhood years are needed for the development of culturally informed family‐based intervention and prevention programs in the Caribbean and other global majority contexts. Accordingly, this study assessed the associations between interparental violence and preschool‐aged children's behavioural difficulties through maternal depressive symptoms in Jamaican families.

## 1 Interparental Violence and Depression and Children's Behavioural Difficulties

Interparental violence not only affects victims but has serious implications for young children's socioemotional functioning. Among studies that have utilized diverse statistical models, interparental violence has consistently been found to have wide‐ranging negative consequences on children that include internalizing (e.g., anxiety, depression, avoidance) and externalizing (e.g., aggression, inattention, conduct problems) behaviour problems, stress and tension, physical injury, and social isolation in different cultural settings (Fong et al. 2019; Jaffee et al. 2002; Goodman et al. 2011; Roopnarine et al. 2023; Yetter 2022). Likewise, two meta‐analyses of studies from high‐income countries showed that maternal depression is associated with internalizing and externalizing behaviour problems, general psychopathology, and higher levels of negative affect and lower levels of positive affect in children (Goodman et al. 2011; Ivanova et al. 2022).

Because interparental violence is a major contributor to the severity of depressive symptoms in women that persists over time (Wright et al. 2015), attempts have been made to determine how interparental violence and depression are associated with children's behavioural difficulties in the same assessment model. Research on the influence of interparental violence on children's behavioural difficulties through maternal depression has produced mixed findings. A handful of studies indicate that maternal depression mediates the link between interparental violence and children's internalizing and externalizing behaviours in high‐income countries (Holmes et al. 2017; Maddoux et al. 2016), but others do not (Graham‐Bermann et al. 2011). Beyond these contradictory findings, it is far from clear how interparental violence and maternal depression in combination are associated with behavioural difficulties in preschoolers among families in global majority contexts such as those in the Caribbean region.

## 2 Caribbean Context

It is estimated that 35% of Jamaican women experience physical assault as victims from a partner during their lifetime (Robinson et al. 2021), and about 7.1% of Jamaican adults are depressed (Abel et al. 2005; Lacey et al. 2016). Research on these two disquieting risk factors has shown that interparental violence has direct negative effects on preschoolers' internalizing and externalizing behaviour problems in Guyana (Roopnarine et al. 2023), and maltreatment and experiences of physical intimate partner violence in childhood are associated with anxiety disorders and suicide ideation in adulthood among Caribbean immigrants in the United States (Lacey et al. 2022). At the same time, maternal depression is related to involved, restrictive, and forceful feeding behaviours in Antigua, Jamaica, and St. Lucia (Wright et al. 2021), to childhood depression among Jamaican school‐aged children (Kirsch et al. 2002), and to internalizing behaviour problems among Guyanese preschoolers (Roopnarine et al. 2023).

These modest attempts to explore associations between family risk factors and childhood development have largely focused on bivariate associations between interparental violence and depression and childhood outcomes or have entered these two risk factors as independent predictors of children's behavioural difficulties in the same assessment model (e.g., Roopnarine et al. 2023). We could not find any studies that assessed the influence of women's experiences of interparental violence and depressive symptoms on children's behavioural functioning in combination in the Caribbean region. In countries like Jamaica, where rates of interparental violence exceed those in other parts of the world, maternal victimization may lead to more elevated depressive symptoms which can then increase levels of behavioural difficulties in children (see Krishnakumar 2021).

## 3 Present Study

This study extends our understanding of the associations between interparental violence and children's anxiety/depression, antisocial behaviours, and peer problems through maternal depressive symptoms in the Caribbean Community (CARICOM) country of Jamaica.

**FIGURE 1:**
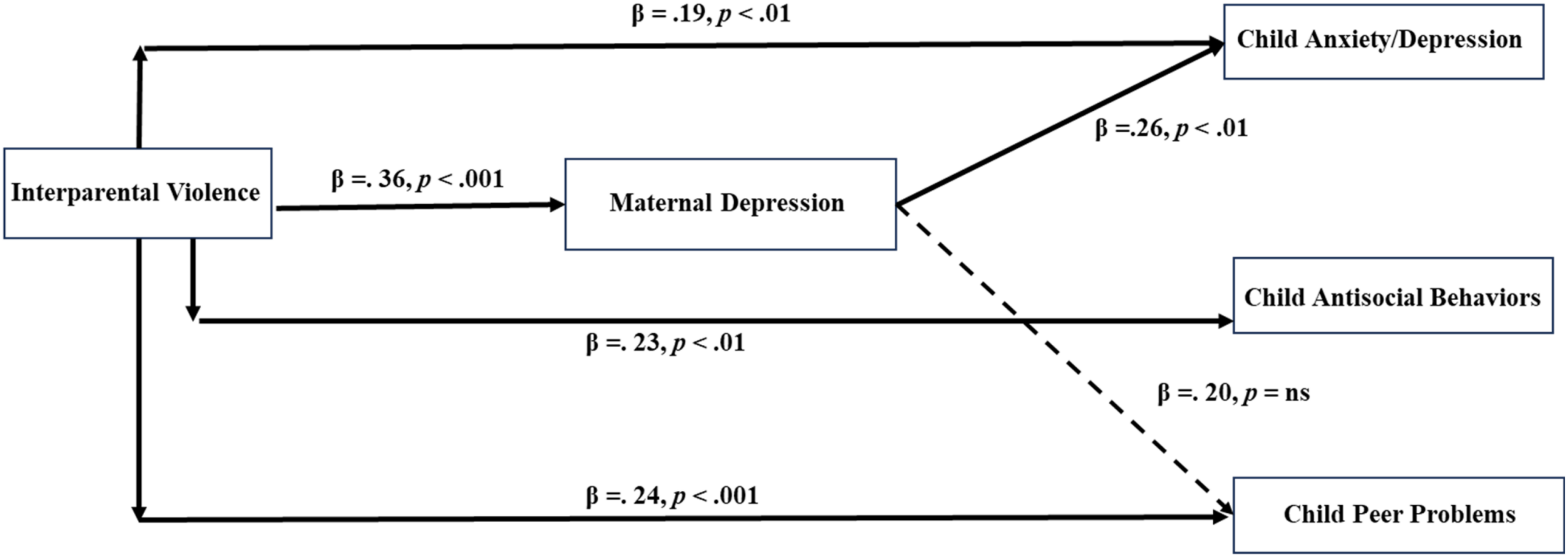
Mediation model linking interpersonal violence and child socioemotional outcomes via maternal depression.

Our conceptual model (see Figure 1) was built as per suggestions within developmental psychopathology and risk and resilience frameworks (Cabrera et al. 2021; Rutter and Sroufe 2000; Masten and Ciccheti 2010) and accompanying research findings on multiple family risk factors and their detrimental effects on childhood development in different cultural communities (Goodman et al. 2011; Roopnarine et al. 2023; Yetter 2022). It is based on the proposition that maternal depression is a viable path through which interparental violence affects children's behavioural functioning. That is, the influences of interparental violence on children's behavioural difficulties may worsen when considered in combination with maternal depressive symptoms. Children's anxiety/depression, antisocial behaviours, and peer problems were chosen as outcome measures in the model because of their connections to children's social functioning within and external to the family and their implications for overall mental health across cultural communities (Achenbach et al. 2016; McWayne et al. 2013). Two hypotheses are tested:

- Jamaican mothers' experiences of interparental violence from men and their depressive symptoms will have direct positive associations with children's behavioural functioning.
- Associations between Jamaican mothers' experiences of interparental violence from men and children's behavioural functioning will be mediated through maternal depressive symptoms.

## 4 Method

### 4.1 Participants

Participants were 152 mothers and their preschool‐aged children residing in the Kingston area of Jamaica. They were recruited through the heads of three preschool programs during the 2021–2022 school year. Most families (92.8%) were from African‐Caribbean backgrounds. The mean age of mothers was 34.08 years (SD = 6.62). Twenty‐two percent (22.4%) of mothers attended high school, 25% attended a technical/trade school, 41.4% attended university and 11.2% had a post‐graduate education. About 64% of families earned over US$600, and 36% earned below US$600 per month. Forty‐four percent of mothers were married, 28.9% were in a common‐law relationship, 14.5% were in a long‐term committed relationship, and 9.9% were single and living apart from their partners. These diverse living arrangements are common across the Caribbean and reflect progressive mating patterns in couple relationships (see Anderson 2021).

There were 71 boys (M = 4.33 years, SD = 0.86) and 81 girls (M = 4.39 years, SD = 0.92). The children attended a range of preschool programs, some privately operated and others supported by the government of Jamaica. Early childhood programs in Jamaica utilize different approaches to education that fall under the umbrella of developmentally appropriate practices, with most stressing basic reading and number skills in laying the foundation for entry into formal schooling. Recruited through the heads of five preschool programs, families were given a brief description of the study, after which written consent was obtained. Preschool programs selected for this study are representative of typical approaches to early education in Jamaica (Kelly‐Williams 2021). About 65% of families who were contacted agreed to participate. Permission to conduct the study was granted by the affiliated University Institutional Review Board in Jamaica.

### 4.2 Procedure

Mothers completed a sociodemographic questionnaire, a depression scale, an intimate partner violence scale and a child behavioural difficulties scale. Mothers received the instruments through the heads of preschool programs. They filled out the instruments in the privacy of their homes and returned them in a sealed envelope to the head teachers of their children's preschool programs.

### 4.3 Sociodemographic Questionnaire

Mothers provided information on ethnicity, educational attainment, income, union/marital status, age, gender, household structure, how many individuals contributed to the household income, and children's age, gender, ordinal position and length of time enrolled in preschool.

### 4.4 Depression Symptoms

Mothers completed the short version of the Centre for Epidemiological Studies–Depression Scale (CES‐D‐12) (Radloff 1977). It contains 12 items (e.g., ‘I felt sad’, ‘I did not feel like eating’, ‘My appetite was poor’, and ‘I could not get going’) that assess depressive symptoms in the previous week. Items are rated on a four‐point Likert‐type scale (*1 = rarely or none of the time to 4 = most or all of the time*). Responses were summed across items to obtain a total score, with higher scores reflecting more depressive symptoms (M = 5.70, SD = 6.32). The CESD‐12 shows good psychometric properties with different ethnic groups in the United States and in Caribbean Community Countries (e.g., Guyana and Trinidad and Tobago; Cronbach's alpha = 0.88) (Roopnarine et al. 2017). Cronbach's alpha for the current study was 0.91.

### 4.5 Interparental Violence

Mothers completed the Revised Conflict Tactics Scales (CTS2) (Straus et al. 1996). From the original scale of 39 items, 20 items that assessed two dimensions of interparental violence were included: *psychological aggression* (eight items: e.g., ‘Threatened to hit or throw something at partner’) and *physical assault* (12 items: e.g., ‘Kicked, bit, or hit partner’). Mothers reported the number of times that they had experienced these dimensions of violence from their partner in the past year (1 = *has never happened*, 2 = *once*, 3 = *twice*, 4 = *3–5 times*, 5 = *6–10 times*, 6 = *11–20 times*, 7 = *more than 20 times*, and 8 = *has not happened in the past year but happened before*). A series of steps were undertaken to create a score of interparental violence. Each of the psychological aggression items was recoded such that 1 = *has never happened* was recoded as 0 = *no psychological aggression*, and scores 2 through 6 were recoded as 1 = *presence of psychological aggression*. If a participant indicated 0 across all 8 psychological aggression items, a score of 0 was given, and if 1 was indicated across any of the 8 psychological aggression items, a score of 1 was given.

Physical assault items were also recoded such that 1 = *has never happened* was recoded as 0 = *no physical assault*, and scores 2 through 6 were recoded as 1 = *presence of physical assault*. If a participant indicated 0 across all 12 physical assault items, a score of 0 was given, and if they indicated 1 across any of the 12 physical assault items a score of 1 was given.

Finally, as has been done with studies on interparental violence in community samples (e.g., Friborg et al. 2015), the dichotomous psychological aggression and physical assault variables were combined into one comprehensive measure of interparental violence for each participant (0 = *no physical or psychological aggression*; 1 = *presence of only one type of violence either physical assault or psychological aggression*; 2 = *presence of both physical assault and psychological aggression*) (M = 0.60, SD = 0.60). It has been suggested in previous studies that the entire scale may be scored dichotomously with categories 1–7 (any occurrence of abuse) indicating prevalence of psychological or physical attacks—all being scored as 1 and with never happened scored as 0. The Cronbach's alpha was 0.95.

### 4.6 Child Socioemotional Difficulties

A version of the Behaviour Problems Index (26 items) was used to assess socioemotional functioning in children. This scale was modified by Peterson and Zill (1986) and shows good internal consistency (Cronbach's alphas between 0.75 and 0.80) in research on different ethnic groups in the United States (Guttmannova et al. 2008; Peterson and Zill 1986; Roopnarine and Yildirim 2017; Zill 1985). Mothers were asked to rate their children's behaviour problems in six domains: anxiety/depression, antisocial, peer problems, dependent, hyperactive and headstrong. Because of our focus on socioemotional functioning, we only used three dimensions of the scale that were of relevance to this study. They included: child anxiety/depression (five items; e.g., Has sudden changes in mood or feelings), child antisocial behaviours (e.g., six items; Bullies, is cruel or mean to others), and child peer problems (two items; e.g., Has trouble getting along with other children) assessed on a four‐point Likert‐type scale (1 = *rarely or none of the time*, 4 = *most of the time*) (child anxiety/depression: M = 1.25, SD = 0.23; child antisocial behaviours: M = 1.22, SD = 0.27; child peer problems: M = 1.13, SD = 0.27). The Cronbach's alpha for the overall scale was 0.69 in this study.

### 4.7 Covariates

Due to their associations with adult intrapersonal and interpersonal relationships and childhood functioning in research on families in different Caribbean countries such as Jamaica, Guyana, Suriname and Trinidad and Tobago (Anderson 2021; Roopnarine and Yildirim 2019; Yildirim and Roopnarine 2017), child gender, child age, maternal education, and parent's marital status were included as covariates in the initial test of our assessment model.

### 4.8 Analytic Strategy

Path analytic approaches were used to test the proposed conceptual model using *Mplus* 8.3 (Muthén and Muthén 1998–2015). To test model fit, we utilized the following statistics: the root‐mean‐square error of approximation (RMSEA) (≤ 0.08; Kline 2005), comparative fit index (CFI) (≥ 0.90; Bentler 1990; Kline 2005), Tucker‐Lewis index (TLI), and the standardized root‐mean‐square residual (SRMR) (≤ 0.08; Hu and Bentler 1999). Bootstrapping techniques (10,000 bootstrap samples) were used to estimate the parameters and to test the significance of the indirect effects. The significance of the indirect effects was evaluated using the 95% bias‐corrected bootstrap confidence intervals (BCBCI) (Mallinckrodt et al. 2006). A 95% confidence interval that excluded zero indicated a significant mediation effect (Mallinckrodt et al. 2006). After running the conceptual model indicated in Figure 1 and examining the significant and nonsignificant pathways, a final mediation model was run after removing the nonsignificant pathways.

## 5 Results

Missing data were limited to a few observations (1% to 2%) and were assessed for randomness using Little's missing completely at random (MCAR) test (Little 1988). The chi‐square statistic and the associated *p* value for Little's MCAR test were not significant, suggesting that data were MCAR and that the missingness could be discounted (χ^2^ (df) = 36.19 (32), *p* = ns). A full‐information maximum likelihood (FIML) approach was used to deal with missing data (Acock 2005). It produces unbiased or less biased parameter estimates and standard errors under both missing at random (MAR) and MCAR assumptions (Enders and Bandalos 2001). The technique utilizes all available data points to provide consistent and asymptotically unbiased parameter estimates and is commonly used in path analysis and structural equation modelling.

Over 40% (41.4%) of mothers reported no incidence of physical assault or psychological aggression, 52.6% reported experiencing either physical assault or psychological aggression and 5.9% reported experiencing both forms of violence. Based on cutoff scores utilized in previous studies to determine the level of depressive symptoms as assessed by the CES‐D‐12 (e.g., Poulin et al. 2005), it was found that 82.9% of mothers had low symptoms, 13.2% had moderate symptoms, and 3.9% had elevated symptoms.

### 5.1 Interrelationship Among Variables

Bivariate correlations among the variables assessed in the conceptual model are presented in Table 1. The correlation coefficients were in the expected direction and provided preliminary support for the conceptual model that was evaluated.

**TABLE 1:** Correlations among constructs used in the study.

	1	2	3	4	5
1. Maternal depression					
2. Interparental violence	0.37***				
3. Child anxiety/depression	0.32***	0.28***			
4. Child antisocial behaviours	0.15	0.24**	0.49***		
5. Child peer problems	0.31***	0.30***	0.46***	0.32***	

*Note:* ***p ≤ 0.001, **p ≤ 0.01, *p ≤ 0.05.

### 5.2 Model

In the initial test of our path model, child gender, child age, maternal education and parent's marital status were included as covariates to determine their associations with children's anxiety/depression, antisocial behaviours, and peer problems. None of these covariates were significant predictors of children's behavioural difficulties and hence were removed from the assessment model. The pathway between maternal depression and child antisocial behaviours was dropped because it was nonsignificant. The test of the new more parsimonious model indicated good model fit (RMSEA = 0.00 (90% CI [0.00, 0.185]), SRMR = 0.01, CFI = 1.00, TLI = 1.00). This model explained 13% of the variance in children's anxiety/depression, 6% in antisocial behaviours, and 13% in peer problems. As can be seen in Figure 1, there were direct significant associations between interparental violence and child antisocial behaviours (β = 0.24, *p* < 0.01) and child peer problems (β = 0.24, *p* < 0.001). There was a significant mediating effect of the association between interparental violence and child anxiety/depression through maternal depression (β = 0.08, 95% CI [0.01, 0.08]). This was a partially mediated pathway as there was a significant direct association between interparental violence and child anxiety/depression (β = 0.19, *p* < 0.01).

## 6 Discussion

Interparental violence victimization by male perpetrators is linked to maternal depression, and in combination, the two can elevate risks to young children (Devries et al. 2013; Fong et al. 2019; Goodman et al. 2011; Neamah et al. 2018; Vu et al. 2016; Yetter 2022). As indicated earlier, research on the joint influence of interparental violence and depression on children's mental health and behavioural functioning is lacking in Caribbean countries. To assist in addressing this gap, this study assessed the associations between interparental violence and children's behavioural difficulties through the mediating function of maternal depressive symptoms in Jamaican families.

### 6.1 Associations Between Interparental Violence and Maternal Depression and Child Outcomes

Our study was guided by tenets within developmental psychopathology and family risk and resilience frameworks that point to the negative impact of multiple family risk factors on childhood development (Cabrera et al. 2021; Rutter and Sroufe 2000; Masten and Ciccheti 2010). Findings indicate that interparental violence victimization and depressive symptoms contribute to behavioural difficulties in preschool‐aged children in Jamaica. Both were associated with children's anxiety/depression, and maternal depressive symptoms partially mediated the associations between interparental violence and children's anxiety/depression but not antisocial behaviours or peer problems. It appears that in the presence of experiences of interparental violence victimization and low to moderate levels of depressive symptoms in Jamaican women, preschoolers are at greater risk for anxiety/depression which can undermine their social interactions with others at home and in the preschool environment. These findings are consistent with those of a recent study on multiethnic families in the United States in which maternal depression partially mediated the associations between interpreter violence and children's internalizing and externalizing behaviour problems (Yetter 2022) and further indicates the detrimental effects of these two risk factors on children's mental health across cultural communities (Devries et al. 2013; Fong et al. 2019; Goodman et al. 2011; Neamah et al. 2018; Vu et al. 2016).

It should be pointed out that the inconsistent associations between interparental violence and children's behavioural difficulties through maternal depression in our study reflect the discrepant nature of findings in the larger body of work in this area. Whereas some studies have shown that depression mediates or partially mediates the association between interparental violence and children's depression and internalizing and externalizing problem behaviours (Holmes et al. 2017; Maddoux et al. 2016), others have failed to do so (Graham‐Bermann et al. 2011). In view of the direct associations between interparental violence and children's antisocial behaviours and problems with peers and that maternal depressive symptoms did not fully mediate associations between interparental violence and children's anxiety/depression, it could be that interparental violence may contribute to children's behavioural difficulties more directly in Jamaican families. Put differently, in the Jamaican cultural context where traditional beliefs about men's roles in the family and society and dominance over women prevail, interparental violence may have more potent effects on children during the vulnerable preschool years. The latter speaks to understanding the impact of interparental violence victimization within male–female roles and family cultural belief systems in Caribbean cultural communities.

Our results bring into focus the concurrent impact of interparental violence on the mental health of mothers and behavioural functioning of children during the early childhood years in Caribbean families. In the main, depression comprises the ability of mothers to meet the emotional and cognitive needs of children which places them at a disadvantage for the promotion of the development of emotional regulation and other social skills in young children.

### 6.2 Limitations and Future Directions

In this study, there are limitations tied to the small and select nature of the sample, the cross‐sectional nature of the data, and the reliance solely on maternal reports of interparental violence, depressive symptoms, and child behavioural difficulties. Future studies would benefit from including fathers from different mating/relationship unions and socioeconomic backgrounds in assessments models to capture how their depressive symptoms work together with perpetration of violence against mothers to influence children's behaviour problems. The use of multiple informants in gathering data, as well as assessments of stability and change in interparental violence and depression over time and their impact on children's behavioural difficulties across age groups, would be prudent as well. Attention to these issues would enable the determination of the combined effects of interparental violence and depression on childhood development more thoroughly, which can then better inform behavioural health and social justice efforts in the Caribbean region.

### 6.3 Implications for Policy and Practice

Because of inadequate mental health infrastructure and counselling services, stigmatized beliefs about accessing mental health counselling, and adverse socioeconomic circumstances, most families and children across the Caribbean region rarely receive treatment for their experiences with interparental violence victimization (see Krishnakumar 2021). These findings could be of use to policymakers, practitioners, governmental and non‐governmental organizations across the Caribbean that focus on mental health and well‐being and social justice issues related to families with very young children who experience family violence victimization. It is also necessary for prevention and intervention programs to consider both interparental violence and depression concurrently because of the spillover effects that one may have on the other in influencing children's mental health. Identifying support systems for mothers and children across the Caribbean region would be of great benefit to families experiencing interparental violence victimization and depression.

## Author Contributions

**Suzette Kelly‐Williams:** investigation, data curation. **Jaipaul L. Roopnarine:** conceptualization, investigation, writing – original draft. **Ambika Krishnakumar:** methodology, formal analysis. **Dickson M. M. Ong'ayi:** formal analysis.

## Funding

This study was supported by funds from an endowed professorship at Syracuse University to the second author.

## Ethics Statement

The current study was approved by the Ethics Review Committee on Research with Human Subjects at Shortwood Teachers' College, Kingston Jamaica.

## Consent

All participants were informed that their participation was voluntary and that the confidentiality of their information would be maintained throughout the study. Completing and submitting the assessment instruments indicated their willingness to participate in the present study; all methods were performed in accordance with relevant guidelines and regulations. All authors have reviewed the final version of the manuscript and consent to its publication.

## Conflicts of Interest

The authors declare no conflicts of interest.

## Data Availability

The data that support the findings of this study are available from the corresponding author upon reasonable request.
